# Abdominal Computed Tomography Scan: A Useful Diagnosis Tool for Early and Delayed Splenic Complications in Malaria

**DOI:** 10.4269/ajtmh.2011.11-0048

**Published:** 2011-07-01

**Authors:** Patrick Imbert, Cécile Ficko, Pierre Buffet, Christophe Rapp

**Affiliations:** Service des Maladies Iinfectieuses et Tropicales; Hôpital d'Instruction des Armées Bégin; Saint-Mandé Cedex, France; E-mail: patrick.imbert@santarm.fr; Service des Maladies Infectieuses et Tropicales; Hôpital d'Instruction des Armées Bégin; Saint-Mandé Cedex, France; Service de Parasitologie Mycologie (Pr. Mazier), & UMR945, INSERM; Paris 6 University; Groupe Hospitalier Pitié-Salpêtrière; Paris Cedex, France; Service des Maladies Infectieuses et Tropicales; Hôpital d'Instruction des Armées Bégin; Saint-Mandé Cedex, France

Dear Sir:

We read with great interest the study on the abdominal computed tomography (CT) scan during acute vivax malaria in South Korea by Kim and others.^1^

In our recent analysis of 55 cases of pathological rupture on the spleen in malaria,^2^ we found that 23 (42%) were caused by *Plasmodium vivax*. We therefore agree with Kim and others that infection with *P. vivax*—less prevalent worldwide than *Plasmodium falciparum*—likely carry a higher risk of splenic complications than infection with *P. falciparum*. Acute splenic enlargement, which is as frequent in *P. vivax* as in *P. falciparum* infection despite lower mean parasitemia in the former,^3^ is likely to be a major determinant of both splenic infarction and splenic rupture in malaria. Splenic enlargement during lethal falciparum malaria likely results from splenic congestion with surface-altered and mechanically altered red blood cells (either infected or uninfected), and with migration or local multiplication of white blood cells.^4^–^6^ Because patients with vivax malaria rarely die, post-mortem data on the spleen in this context are very limited.^7^

The mechanistic connections between malaria, splenic infarction, and pathological splenic rupture are not clearly established. Although splenic infarction leads to splenic rupture in a small number of patients, 53 of the 55 malaria patients with pathological splenic rupture had no obvious signs of pre-existing infarction.^8^

Not least, pathological rupture of the spleen and other splenic complications may occur well beyond the acute phase of the malaria attack (median time from fever onset to rupture: 5 d, range: 0–37; median time from malaria diagnosis to rupture: 0 d, range: 0–31).^2^ Because Kim and others excluded patients in whom the CT scan had been performed more than 3 days after the diagnosis of malaria, a proportion of splenic complications was likely to be ignored in their study.^1^ Clinicians should be aware of the (small but life-threatening) risk of a delayed splenic rupture after a malaria attack.
